# Heme Scavenging and Delivery: The Role of Human Serum Albumin

**DOI:** 10.3390/biom13030575

**Published:** 2023-03-22

**Authors:** Giovanna De Simone, Romualdo Varricchio, Tommaso Francesco Ruberto, Alessandra di Masi, Paolo Ascenzi

**Affiliations:** 1 Department of Sciences, Section of Biomedical Sciences and Technologies, Roma Tre University, 00146 Roma, Italy; 2 Centro Linceo Interdisciplinare Beniamino Segre, Accademia Nazionale dei Lincei, 00165 Roma, Italy; 3 Accademia Nazionale dei Lincei, 00165 Roma, Italy

**Keywords:** catabolism, CD71 receptor, heme, heme export, heme import, heme scavenging, hemoglobin, hemopexin, hemophore, human serum albumin

## Abstract

Heme is the reactive center of several metal-based proteins that are involved in multiple biological processes. However, free heme, defined as the labile heme pool, has toxic properties that are derived from its hydrophobic nature and the Fe-atom. Therefore, the heme concentration must be tightly controlled to maintain cellular homeostasis and to avoid pathological conditions. Therefore, different systems have been developed to scavenge either Hb (i.e., haptoglobin (Hp)) or the free heme (i.e., high-density lipoproteins (HDL), low-density lipoproteins (LDL), hemopexin (Hx), and human serum albumin (HSA)). In the first seconds after heme appearance in the plasma, more than 80% of the heme binds to HDL and LDL, and only the remaining 20% binds to Hx and HSA. Then, HSA slowly removes most of the heme from HDL and LDL, and finally, heme transits to Hx, which releases it into hepatic parenchymal cells. The Hx:heme or HSA:heme complexes are internalized via endocytosis mediated by the CD91 and CD71 receptors, respectively. As heme constitutes a major iron source for pathogens, bacteria have evolved hemophores that can extract and uptake heme from host proteins, including HSA:heme. Here, the molecular mechanisms underlying heme scavenging and delivery from HSA are reviewed. Moreover, the relevance of HSA in disease states associated with increased heme plasma concentrations are discussed.

## 1. The Double Face of the Heme

Heme is an iron-containing porphyrin that constitutes the prosthetic moiety of heme proteins [1]. Heme is synthesized through a series of reactions that take place in the mitochondrion and in the cytoplasm of all eukaryotic cells [2]. Daily, human erythroid cells synthesize 75% of the total body heme (~300 mg of heme/day), which is incorporated in hemoglobin (Hb), whereas hepatocytes produce ~50 mg of heme/day, which represents the central site of catalases, cytochrome P450, cytochrome B5, myoglobin (Mb), cytochrome *c*, and other mitochondrial cytochromes [1,2]. Heme is the catalytic center of hemoproteins exerting several crucial biological functions such as oxygen sensing, cell respiration and metabolism, growth, self-renewal, and differentiation. Indeed, hemoproteins include: (*i*) Hb and Mb that allow oxygen transport and storage; (*ii*) cytochromes, which are involved in cell respiration in mitochondria (i.e., cytochrome *c*, cytochrome *c* oxidase (COX), and cytochrome reductase); (*iii*) drug-metabolizing cytochromes P450; (*iv*) enzymes (*e.g.*, catalases, peroxidases, guanyl cyclases, nitric oxide synthases, histidine kinases, cyclic nucleotide phosphodiesterases); and (*v*) heme-responsive transcription factors with basic helix–loop–helix (bHLH) DNA-binding domain motif. Of note, heme is also part of cyanocobalamin (also named vitamin B12) [1,3,4,5]. 

In contrast to the important heme-based biological functions of hemoproteins, the free heme, defined as labile heme pool, has toxic properties that originate from its hydrophobic nature and from the presence of the Fe-atom [2]. The labile heme pool is derived either from newly synthesized heme that has not yet been incorporated into hemoproteins or from heme that has been released from hemoproteins under oxidative conditions. The labile heme pool acts as an “alarmin” [6] as it is sensed by pattern recognition receptors such as the Toll like receptor [7], and NACHT, LRR, and PYD domains-containing protein 3 (NALP3) [8]. The labile heme pool may increase after extracellular heme overload, increased heme synthesis, accelerated hemoprotein breakdown, impaired incorporation into apo-proteins, and impaired heme-oxygenase (HO) activity [1,9,10]. 

Free heme is an abundant source of ferrous iron (Fe(II)) that can participate in the Fenton reaction, a process during which reactive oxygen species (ROS) are produced. Free heme is extremely toxic for cells. Indeed, heme (*i*) intercalates biologic membranes altering lipid bilayers; (*ii*) is strongly pro-inflammatory, inducing the recruitment of leukocytes, platelets, and red blood cells (RBCs) to the vascular endothelium; (*iii*) oxidizes low-density lipoproteins (LDLs); and (*iv*) inactivates nitric oxide, thus impairing vascular functions [1]. Furthermore, ROS produced by the heme-driven Fenton reaction can (*i*) damage lipid membranes, proteins, and nucleic acids; (*ii*) activate cell signaling pathways and oxidant-sensitive pro-inflammatory transcription factors; (*iii*) alter protein expression; and (*iv*) perturb membrane channels [1,2,5]. All these events contribute to promoting cell death [2,5].

Here, the molecular mechanisms underlying the role of human serum albumin (HSA) in heme scavenging and delivery are discussed, highlighting the competition between mammalian cells and pathogens in heme up-taking. 

## 2. Regulation of Heme Levels

**Figure 1:**
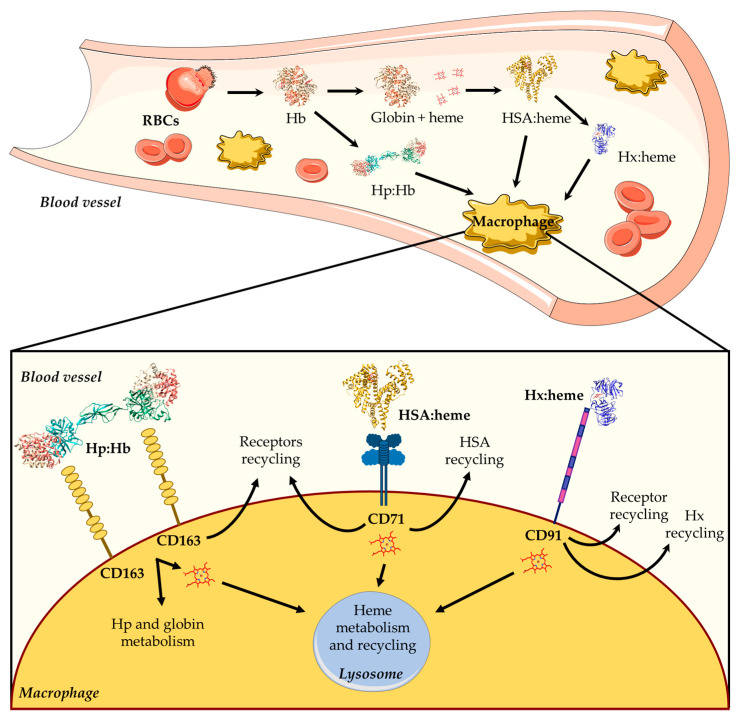
Overview of heme scavenging in blood vessels. The intravascular hemolysis of red blood cells (RBCs) induces the release of hemoglobin (Hb), which is scavenged by high-density lipoproteins (HDLs), low-density lipoproteins (LDLs), human serum albumin (HSA), and hemopexin (Hx). First, the free heme binds to HDL and LDL, then it moves to HSA, and lastly it is transferred to Hx. The HSA:heme complex [31] is internalized by the CD71 receptor, whereas the Hx:heme complex [32] binds to the CD91 receptor and is then moved into macrophages. The figure has been partially generated through the website Servier Medical Art licensed under a Creative Commons Attribution 3.0 imported license. The three-dimensional structures of Hb (PDB ID: 1JY7) [33], Hp:Hb (PDB ID: 4F4O) [34,35], HSA:heme (PDB ID: 1N5U) [31], and Hx:heme (PDB ID: 1QJS) [32] complexes have been drawn using UCSF-Chimera [36].

Hb is the main blood hemoprotein responsible for O_2_ delivery into the circulatory system, also playing a key role in ROS and reactive nitrogen species (RNS) detoxification [11,12,13]. Although Hb is normally confined to RBCs, low levels of extra-erythrocytic Hb and free heme in the plasma may be due to physiological phenomena associated with intravascular hemolysis, which occur during the destruction of senescent erythrocytes and the enucleation of erythroblasts [11,14,15]. Because of the potential extracellular toxicity of free heme, its concentration is tightly controlled to maintain cellular homeostasis and to avoid pathological conditions. To this purpose, mammals have developed different systems able to scavenge either Hb (i.e., haptoglobin (Hp)) or free heme (i.e., high-density lipoproteins (HDL), low-density lipoproteins (LDL), hemopexin (Hx), and human serum albumin (HSA)) [1,2,16,17,18,19] (Figure 1). During the physiological turnover of RBCs, the small fraction of free extracellular Hb released into the plasma (~10 %) [20] is captured by Hp and transported to reticulo-endothelial macrophages located in the liver and in the spleen, which represent the main sites for the clearance of aged and damaged RBCs. Then, the Hp:Hb complex is captured by the Hp scavenger receptor (i.e., CD163) and is internalized [1,17] (Figure 1). Following RBCs phagocytosis or Hp-mediated Hb internalization, Hb is degraded and the heme is either recycled for de novo erythropoiesis or catabolized. 

### 2.1. Heme Scavenging in Plasma

Heme scavenging by HDL, LDL, HSA, and Hx provides protection against free heme oxidative damage, limits access by pathogens to heme, and contributes to iron homeostasis by recycling the heme iron. In the first seconds after heme appearance, more than 80% of this macrocycle binds to HDL and LDL, and only the remaining 20% binds to Hx and HSA. In particular, HSA slowly removes most of the heme from HDL and LDL and transfers it to Hx, which finally releases the macrocycle into hepatic parenchymal cells via endocytosis mediated by the CD91 receptor [1,2,11,16,17,18,21] (Figure 1). Of note, free heme binds to HSA (*K*_d_ ~ 1.0 × 10^−9^ M) with a lower affinity compared with Hx (*K*_d_ < 10^−9^ M) [11].

Although HDL and LDL are the most oxidatively intolerant plasma components, they bind heme with a high affinity (*K*_d_ ranging between 10^−11^ M and 10^−10^ M) and at a faster rate than HSA and Hx. Of note, the kinetics of heme release from HDL and LDL is faster than the kinetics of heme-induced lipoprotein oxidation [18,22,23]. 

RBCs clearance and extracellular Hb scavenging are relatively modest events under steady-state conditions, but are drastically enhanced in hemolytic disorders (e.g., anemia, vasculopathy, endothelial dysfunction, and infections), when high levels of extracellular Hb and heme ultimately lead to the saturation and depletion of Hp and Hx scavenging systems [24], causing heme-mediated oxidative damage to tissues [1,2,25]. When the buffering capacity of plasma Hp is exceeded, hypoxia induces the quick oxidation of Hb to methemoglobin (metHb) [26]. Cell-free Hb that becomes oxidized or denatured prior to clearance is prone to release free heme [11,15,27]. As the Hx plasma concentration (~1.5 × 10^−5^ M) is about two orders of magnitude lower than that of HSA (~7.5 × 10^−4^ M) [11], the release of a massive quantity of heme reduces the bioavailability of Hx, and consequently HSA acts as the main heme scavenger [28,29]. Indeed, in patients with hemolytic disorders, the plasmatic level of the HSA:heme complex increases from ~1.0 × 10^−6^ M in physiological conditions, to ~4.0 × 10^−5^ M [23,24,30]. 

### 2.2. Regulation of the Intracellular and Extracellular Heme Levels

The regulation of the extracellular and intracellular heme levels is crucial for preventing pathological iron or heme accumulation. Heme synthesis and degradation are inhibited and induced, respectively, by heme itself. Heme production is regulated differently in erythroid and non-erythroid cells. In non-erythroid cells, heme reduces its own production by (*i*) decreasing the activity of ALA Synthase 1 (ALAS1), the rate-limiting enzyme in heme biosynthesis, and (*ii*) increasing its catabolism [1]. On the contrary, in erythroid cells, heme functions as a positive feedback regulator, promoting its synthesis and inhibiting its breakdown. Importantly, the amount of newly synthesized heme must match the rate at which it is incorporated into apo-hemoproteins. This balance is achieved through the regulation of both heme and apo-hemoprotein synthesis. Indeed, heme can induce the expression of various apo-hemoproteins, such as Hb, myoglobin, neuroglobin, cytochromes. This evolutionary mechanism helps preventing the toxic accumulation of intracellular heme and ensures that the amount of heme synthesized is properly balanced with the amount incorporated into hemoproteins or eventually catabolized [1]. 

The physiological degradation of heme occurs in a tightly controlled manner through the activity of HO, which cleaves heme in the presence of NADPH + H^+^ and O_2,_ resulting in the production of carbon monoxide (CO), Fe(II), and biliverdin IX. Fe(II) is either bound to ferritin, which represents the main intracellular iron storage protein, or exported into the bloodstream by ferroportin. Biliverdin IX is degraded by a NADPH-dependent reductase into bilirubin IX, which is finally excreted in the bile and urine [17,19,25,37,38,39]. Biliverdin, bilirubin, HO-1, and CO all display important antioxidant properties [40]. The major site of heme breakdown is the liver, although spleen, brain, and erythropoietic system are also important heme catabolic organs [1,21,39]. When not catabolized, intracellular heme must be either incorporated into apo-proteins or expelled out from cells. The most characterized heme exporters are the Feline Leukemia Virus Subgroup C Receptor 1a (FLVCR1a) [41] and the ATP-binding cassette sub-family G 2 (ABCG2) [42].

On the contrary, when heme is requested for incorporation into apo-proteins, it can be either newly synthesized or imported from the extracellular space. Heme is ubiquitously synthesized through eight enzymatic reactions that take place between the mitochondria and the cytoplasm. The rate-limiting step of this process is represented by the first reaction that is catalyzed by the ALAS1 enzyme, which condensates glycine with succinyl CoA to form aminolevulinic acid (ALA) [1,40]. 

To date, the only known proteins with a well-established function as heme importers are (i) the Heme-Responsive Gene 1 (HRG1) [43,44] and the FLVSCR 2 (FLVCR2), which are expressed ubiquitously [45], and (ii) the Heme Carrier Protein 1/Proton-Coupled Folate Transporter (HCP1/PCFT), which is expressed in the duodenum, liver, kidney, spleen, and placenta, and to a minor extent in the colon, rectum, ileum, jejunum, cecum, and testis [40,46].

## 3. Heme Scavenging from HSA

HSA is synthesized in the hepatocytes at the rate of ~0.7 mg/hour/gram of liver (i.e., at ~10–15 g/day) and is then released into the plasma where its concentration ranges from 5.3 × 10^−4^ M to 1.0 × 10^−3^ M. The rate of HSA synthesis depends mainly on (*i*) the blood oncotic pressure, as the HSA concentration is detected by osmoreceptors in the hepatic interstitium, (*ii*) hormonal stimuli, (*iii*) nutrition, and (*iv*) inflammation [47,48,49]. 

HSA is a monomeric globular protein of 585 amino acids (molecular weight of ~66 kDa) composed of 67% α-helix without β-sheet, and organized in three domains (i.e., I, II, and III) encompassing amino acids 1–195, 196–383, and 384–585, respectively. Each domain includes 10 helices organized in subdomains A and B that are built from six and four α-helices, respectively, connected by a long loop [16,30,50,51]. HSA binds up to nine equivalents of fatty acids (FAs), its primary physiological ligands, at sites FA1 to FA9 [30,52]. HSA also binds heme, metal ions, hormones, and nucleic acids. it affects pharmacokinetics of many drugs, renders potential toxins harmless, accounts for most of the antioxidant capacity of human plasma, and displays (pseudo-)enzymatic activities [16,52,53,54,55].

Heme binding to HSA is a simple process, with *K*_(heme)_ = 1.3 × 10^−8^ M, *k*_on (heme)_ = 7.4 × 10^5^ M^−1^ s^−1^, and *k*_off (heme)_ = 9.6 × 10^−3^ s^−1^ [56]. Heme binds HSA at the center of subdomain IB (i.e., at the FA1 site). First, heme binds reversibly to His146 at the surface of HSA to generate an intermediate complex; then, the macrocycle binds to the Tyr161 residue placed within subdomain IB [57]. Interestingly, the HSA:heme complex displays globin-like catalytic properties, including peroxynitrite scavenging functions as well as catalase and peroxidase activities [16,58,59].

**Figure 2:**
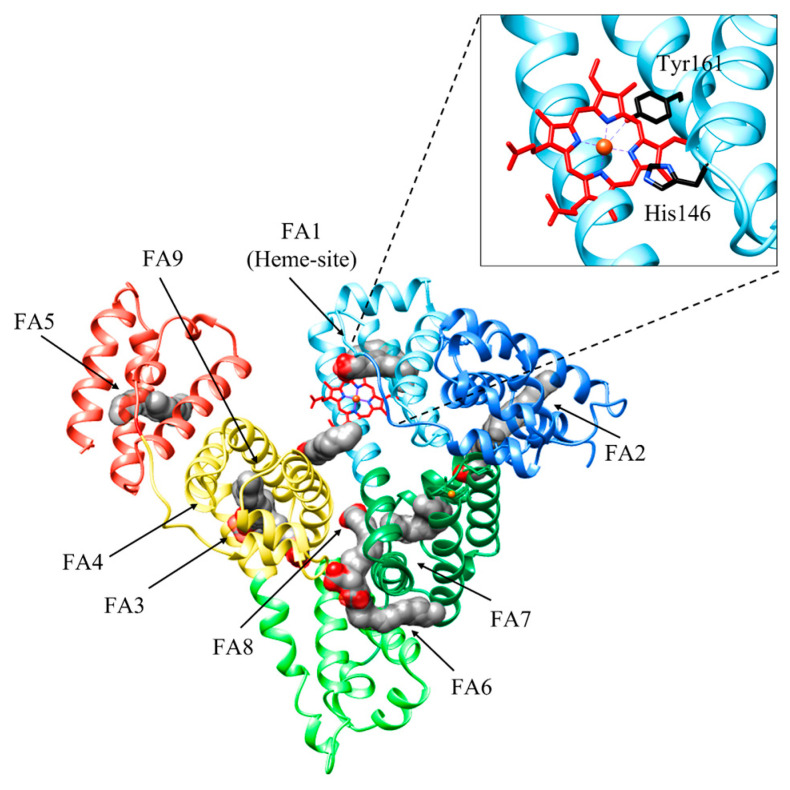
Three-dimensional structure of HSA bound to fatty acids (FAs) and heme. HSA is organized in three domains: domain IA (light blue), domain IB (sky blue), and domain IA (forest green); domain IIA (light green); and domain IIIA (yellow) and domain IIIB (coral). HSA binds up to nine equivalents of FAs, its primary physiological ligands, at sites FA1 to FA9. FAs are rendered as space-fill (gray), whereas the heme is rendered as sticks (red). His141 and Tyr146 residues coordinating the heme-Fe atom are shown in black. The subdomains of HSA are rendered with different colors. The picture has been drawn using the UCSF Chimera package [36].

Heme binding to HSA is modulated by drug binding to the FA2 and FA7 sites, and vice versa, through competitive and allosteric mechanisms. Indeed, drugs binding to the FA1 site (e.g., rifampicin, isoniazid, and fipronil) directly impairs heme recognition. Moreover, warfarin binding to the FA7 site (i.e., Sudlow’s site I) decreases by about one order of magnitude the values of *K*_(heme)_ and *k*_on (heme)_ for heme binding to HSA at the FA1 site. In turn, heme binding to HSA inhibits drug binding to the FA2 and FA7 sites [60,61,62]. Upon drug binding to either the FA2 or the FA7 sites, the reorientation of the Glu131-Arg145 α-helix occurs causing a shift in the Phe149-Tyr150 dyad and the Arg257 residue next to the FA1 cavity. This reduces heme affinity, induces the hexa-coordination of the metal center by the His146 residue, and inhibits heme-based reactivity [30,62,63] (Figure 2). Of note, drugs binding to HSA:heme inhibits the heme-based detoxification of ROS and RNS [16,56,64,65,66,67,68]. As heme scavenging from HSA is a transient process, the heme-based catalytic properties of HSA represent a case of “chronosteric effects” [66].

The modulation of heme binding to HSA by drugs may be relevant in pharmacotherapy management. Indeed, an increase in heme levels under pathological conditions (e.g., severe hemolytic anemia, crash syndrome, and post-ischemic reperfusion) increases the drug plasma concentration and induces the release of HSA-bound drugs with the consequent patient intoxication [16,30,62,66,69,70,71,72,73,74,75,76,77]. In turn, high levels of heme-albumin due to hemolytic events or pathological states characterized by low HSA levels (i.e., hypoalbuminemia, [HSA] < 5.3 × 10^−4^ M) may cause a lower availability of circulating albumin. This determines an increase in the unbound fraction of drugs, resulting in a lower efficacy [72]. However, this does not necessary result in potential adverse effects, because many drugs can bind not only HSA, but also lipocalins (e.g., α-1-acid glycoprotein (AGP) and retinol-binding protein 4 (RPB4)) [30,72].

## 4. HSA: Heme Complex Internalization

Upon secretion from hepatocytes, HSA enters the circulation and translocates to the extracellular space through the pores of sinusoidal or fenestrated endothelium cells of the liver, pancreas, small intestine, and bone marrow [78,79]. HSA can cross the endothelium via active transcytotic mechanisms, including receptor-mediated processes [80]. These receptors can selectively recognize the native or conformationally modified HSA (e.g., gold-labeled HSA, formaldehyde- or maleic anhydride-treated HSA [80,81]). To date, eight membrane-associated HSA binding proteins have been described: albondin/glycoprotein 60 (gp60) [82], glycoprotein 18 (gp18) [83], glycoprotein 30 (gp30) [83], neonatal Fc Receptor (FcRn) [84], heterogeneous nuclear RiboNucleoProteins (hnRNPs) [85], calreticulin [85], cubilin [86,87], megalin [86,87], and Secreted Protein Acidic and Rich in Cysteine (SPARC) [88].

To date, very little is known regarding the HSA:heme internalization mechanisms. Recently, it has been suggested that CD71 (also known as Transferrin Receptor 1, TfR1) acts as a specific cellular receptor for the HSA:heme complex [89]. CD71 is ubiquitously expressed, is bound to two Fe(III) atoms (Tf:Fe(III)_2_), and internalizes transferrin (Tf), [90,91]. CD71 is a homodimeric type II transmembrane protein composed of a small cytoplasmic domain, a single-pass transmembrane region, and a complex extracellular domain. Each monomer of the ectodomain is composed of (*i*) a protease-like domain that is in contact with the cell membrane, (*ii*) a helical domain that comprises the homodimer interface, and (*iii*) an apical domain. The ectodomain binds several proteins. Indeed, the CD71 basal portion, composed of the protease-like and the helical domains, recognizes Tf; the dimer interface region forms a complex with the hereditary hemochromatosis factor (HFE); the upper part of the apical domain interacts with ferritin as well as with *Arenaviruses* and with the *Plasmodium vivax* invasion protein PvRBP2b1, which exploit CD71 for cell invasion [92].

HSA:heme binding to CD71 allows for complex internalization and represents an alternative source of iron to Tf:Fe(III)_2_. This implies that HSA plays a role in providing iron to cells, which is fundamental to sustain vital processes such as cell metabolism and proliferation [89]. Both the HSA:heme complex and Tf-Fe(III)_2_ recognize the basal portion of the CD71 ectodomain. The *K*_d_ value for HSA:heme binding to CD71 is lower than that of Tf:Fe(III)_2_ at physiological pH, depending on the species and on the tissues (*K*_d(HSA:heme)_ = 7.5 × 10^−7^ M; *K*_d(Tf-Fe(III)2)_ ~ 10^−8^ M) [89]. The CD71/HSA:heme recognition mechanism appears to be species specific; indeed human CD71 is unable to recognize the bovine serum albumin:heme complex [89]. Upon internalization, the HSA:heme complex can be used as a Fe(III) source by primary human T cells, as well as by immortalized cell lines [89]. Once HSA:heme is internalized, the isoform 1 of HO (i.e., HO-1) is pivotal to utilize heme as a Fe source. Indeed, while supplementation of serum-free medium with HSA:heme supports the growth of lymphoblastoid cells expressing wild-type HO-1, the supplementation is ineffective in HO-1 deficient lymphoblastoid cells. Furthermore, the proliferation of primary human T cells in the presence of HSA:heme is inhibited by the Tin protoporphyrin HO-1 inhibitor [89].

It is noteworthy that the HSA:heme complex shows peroxidase activity, which is a well-known antimicrobial mechanism of the human innate immune response [93,94]. As some viruses causing hemorrhagic fever (e.g., *Arenavirus*, *Machupo* virus) use the CD71 receptor to enter human cells, high levels of HSA:heme may exert a protective function towards CD71-mediated virus entry [89].

## 5. Functional Aspects of the HSA-Dependent Heme Internalization

The differentiation of macrophages of the reticuloendothelial system is modulated by HSA:heme complex internalization. This is pivotal for the modulation of the inflammatory response in patients showing either acute or chronic heme release in the plasma [95]. Heme, and specifically its iron moiety, promotes pronounced changes in macrophages towards an M1-like proinflammatory phenotype. The classically activated M1 macrophages, induced by microbial agents and proinflammatory T-helper (Th) 1 cytokines, can exert inflammatory functions and bactericidal activity, and can induce high levels of proinflammatory cytokines, ROS, and RNS. On the contrary, the alternatively activated M2 macrophages, generally induced by Th2 cytokines, exert immunoregulatory functions, support pathogen clearance, and are involved in cell growth control, matrix remodeling, angiogenesis, and tissue repair [95].

Notably, Hx reduces heme accumulation in macrophages, thus preventing heme-induced proinflammatory phenotypic switching from M2 to M1, both in vitro and in vivo [95,96]. The administration of exogenous Hx in an experimental model of hemolysis and in a mouse model of Sickle cell disease is beneficial in counteracting the heme-driven proinflammatory status of macrophages [97]. On the contrary, heme levels are significantly increased in bone marrow-derived macrophages treated with HSA:heme compared with those treated with Hx:heme. Furthermore, monocyte/macrophage-like cells treated with HSA:heme accumulate higher levels of heme, show increased levels of H- and L-ferritin chains, ferroportin, and HO, and display enhanced HO-1 activity compared with Hx:heme treated cells. In addition, ROS production and IL-6 and TNFα expression increased in cells treated with HSA:heme rather than those supplemented with Hx:heme. These findings indicate that HSA allows for a delivery rate of heme into macrophages that is significantly higher than that of Hx, thus playing a key role in driving the transition from M2 to M1 macrophages [95].

## 6. HSA, Heme, and COVID-19

The SARS-CoV-2 virus binds to Hb and causes heme release, resulting in impaired O_2_ supply and ROS generation. In turn, this causes increased oxidative stress, hypoxia, and potential cardiac injury (e.g., heart attack and cardiac arrest) [98,99,100]. Recently, it has been reported that ORF1Ab, ORF3a, and ORF10 SARS-CoV-2 viral proteins can coordinately uptake the heme localized in the β chains of Hb. Both oxygenated and deoxygenated Hb can be attacked, but the latter is more sensitive to the virus [100]. Indeed, COVID-19 patients showed increased heme plasma levels [101], comparable to those reported under hemolytic conditions (~2.0 × 10^−5^ M) [102]. To properly buffer high levels of the labile heme pool and to counteract the consequent inflammatory response, the production of heme-scavenging proteins such as Hx and HSA is increased [98,103]. Accordingly, hypoalbuminemia is significantly correlated with COVID-19 progression and severity as a predictive index of the disease outcome, independently from patient age and morbidity [98,104,105,106,107,108].

## 7. Role of HSA in Heme Uptake by Bacteria and Fungi

Heme also constitutes a major iron source for pathogens such as bacteria and fungi. To overcome the scarcity of free iron in the animal host, pathogens have developed systems to extract and uptake heme from host proteins. The strategies of iron acquisition are (*i*) the activation of the secondary metabolism for the production of small iron-chelating compounds, called siderophores, and (*ii*) specialized secreted and/or membrane-bound hemophores that are able to acquire both free heme and heme bound to Hb, Hp:Hb, Hx:heme, HSA:heme, and Mb [29,66,109,110,111] in order to deliver heme to a specific outer membrane receptor expressed on the pathogen membrane (see [66], and the references therein). Both siderophores and hemophores contribute to the virulence of many pathogens such as *Bordetella*, *Haemophilus*, *Brucella*, *Vibrio*, *Streptococcus*, and *Staphylococcus*, and many other Gram-positive and Gram-negative species. Interestingly, hosts characterized by high levels of free heme are generally more susceptible to infection. This implies that hemophores preferentially uses the labile free heme compared with the bound fraction available in the host [112].

HSA promotes heme utilization in fungi belonging to the *Candida* species. This HSA stimulatory activity could reflect the solubilization of free heme that otherwise would aggregate in the solution, with a consequent reduction in its effective concentration [111]. HSA-induced heme utilization was reported for albumin concentrations as low as 5.0 × 10^−6^ M, which means more than two orders of magnitude lower than the HSA concentration in human plasma. This implies that HSA tissue concentrations (ranging from 6.5 × 10^−2^ M to 2 × 10^−1^ M) could be sufficient to provide heme-iron acquisition for fungi that have penetrated tissues [111,113]. As the affinity of HSA for heme might be in the same range as that of bacterial surface receptors for heme, it is likely that heme bound to HSA is recognized by heme/Hb receptors, and then it is passively transferred to them from HSA [111].

## 8. Clinical Use of HSA in Hemolytic Diseases

From a clinical perspective, the hypoalbuminemic condition is correlated with an increased risk of mortality in several diseases in which hemolytic events occur (e.g., malaria, general systemic inflammation, sepsis, cirrhosis, splenomegaly, portal hypertension, lupus erythematosus, and infectious diseases) [98,104,105,106,107,108,114,115]. A significant association between increased hemolytic markers and both albuminuria and glomerular hyperfiltration has been reported in patients with severe forms of sickle cell anemia and thalassemia [116]. Overall, labile heme exerts pro-inflammatory, vasoactive, and cytotoxic effects that can contribute to the pathogenesis of hemolytic conditions [117,118,119,120,121,122,123,124].

Hepatic dysfunction occurs frequently during sepsis, whose pathogenesis is driven by an inadequate response of the host to infection, leading to dysregulation of iron metabolism and the buildup of labile heme. Of note, heme and Hb scavenging reduces disease severity. The observation that the administration of 4% albumin reduces oxidative stress, mortality, and endothelial and kidney dysfunctions in mice subjected to endotoxemia, which is induced by labile heme, supports the protective role towards sepsis of HSA [125,126], by reducing heme-mediated in vitro cytotoxicity and in vivo heme-mediated vasoconstriction [115]. 

In a prospective cohort study of 116 septic patients, it was found that those with low concentrations of HSA had a poorer outcome. Furthermore, a subgroup analysis of patients with severe sepsis enrolled in the ALBIOS trial showed that administering HSA might improve survival [127]. Moreover, the results of the study validate the beneficial effects of administering HSA during severe sepsis, which includes increasing the distribution of fluids within the intravascular compartment. Indeed, HSA may act as a scavenger of nitric oxide, leading to peripheral vasodilatation during sepsis [127]. A recent retrospective study conducted on 2829 patients hospitalized between January 2013 and April 2018 with a diagnosis of sepsis/septic shock showed that the use of HSA within 24 h of hospital admission was associated with a shorter time to discharge and a higher rate of discharge with clinical stability, suggesting an improvement in healthcare resource utilization among patients [128]. 

Malaria is a severe disease caused by parasites of the *Plasmodium* genus. As part of its life cycle, the parasite consumes Hb from RBCs, leading to the release of heme. At least one third of malaria patients are hypoalbuminemic. Moreover, an association between low serum albumin levels and both a longer parasitemia time and a higher incidence of cerebral malaria has been found [129]. Interestingly, a randomized trial comparing HSA and saline in children with malaria demonstreated that the mortality rate was significantly lower among patients who received albumin than among those who received saline (3.6% vs 18%; 95% CI 1.2–24.8; *p* = 0.013) [130]. Similarly, a controlled trial in malaria children demonstrated that those who received HSA underwent mortality in the coma with a significantly lower incidence (1/25; 4%) compared with patients who received the colloid Gelofusine (6/23; 26%); however, the underpowered sample size did not allow for solid inferences [131]. As heme toxicity contributes to cerebral malaria pathogenesis, a specific neuroprotective effect of albumin can be hypothesized [131]. However, no further evidence based on clinical trials is still available. 

HSA is also of primary importance in the binding and detoxification of bilirubin, the end product of heme catabolism, whose concentration increases during hemolytic events [132]. Unbound bilirubin can cross the blood−brain barrier and cause neurotoxicity [133,134]. The administration of HSA has been proven to be very beneficial in hyperbilirubinemia, which is a common condition in the neonatal period. Indeed, hyperbilirubinemic infants treated with HSA showed reduced levels of circulating unbound bilirubin, thus decreasing the incidence of complications such as fever, allergic reactions, and encephalopathies [135]. Of note, medications that interfere with HSA:bilirubin binding or that inhibit the p-glycoprotein, increase the risk of acute bilirubin encephalopathy [136].

HSA could also be used as a therapeutic adjuvant in major post-operatory complications such as kidney’s ischemia reperfusion injuries, in which high levels of free heme in the kidney are correlated with inflammation after organ transplants [137,138]. In a study involving a mouse model of kidney ischemia, it has been observed that HSA was able to reduce the release of pro-inflammatory cytokines and the expression levels of complement receptors in the renal tissue [138]. In this regard, future studies will be required to develop clinically applicable therapies to reduce the effects of free heme in ischemic organs, which, in turn, may result in more favorable post-transplant outcomes.

## 9. Conclusions and Perspectives

**Figure 3:**
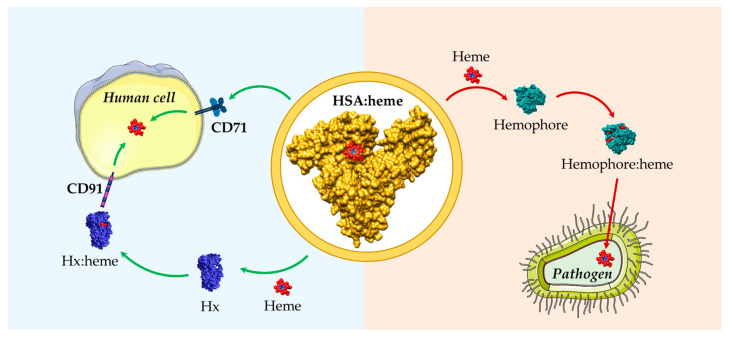
Schematic representation of heme scavenging and delivery by HSA in both human cells and pathogens. HSA can either act as a heme scavenger in human blood reducing the free heme labile concentrations or as a heme donor to pathogens. HSA:heme (PDB ID: 1N5U) [31], Hx:heme (PDB ID: 1QJS) [32], and the *C. albicans* Csa2 hemophore in complex with heme (Csa2:heme, PDB ID: 4Y7S) [110] have been drawn with UCSF-Chimera [36]. The figure has been partially generated using the website Servier Medical Art, provided by Servier, licensed under a Creative Commons Attribution 3.0 unported license.

HSA binds free heme with a high affinity, contributing to its scavenging and to the maintenance of cellular homeostasis, and avoiding free heme-related toxicity. Heme scavenging by HSA could also modulate the bioavailability of this macrocycle to pathogens as an iron source. Therefore, HSA plays a key role in regulating heme metabolism, influencing both eukaryotic and prokaryotic cell growth (Figure 3). However, the overall ability of HSA to facilitate heme-Fe utilization by pathogens needs to be further clarified.

The high plasma concentration of HSA (~10^−4^ M) and the high endogenous and exogenous ligands affinity for HSA (*K*_d_ < 10^−4^ M) implies that the fraction of free ligand(s) in the plasma is negligible compared with that bound to HSA. This implies that ligand internalization may occur only by an HSA-dependent mechanism, either through the uptake of the HSA:ligand complex to cell receptors/channels or by ligand transfer from HSA to cell surface proteins. In the last case, a transient trimeric complex built by the HSA:ligand:receptor should occur.

The transfusion of donor blood has become a common and routine practice. However, the requirement for an enhanced level of safety has a significant cost, and blood transmitted infection remains a challenging problem. Additionally, donor blood transfusions necessitate crossmatching and compatibility testing to prevent a hemolytic reaction in the recipient, and the purified RBCs must be stored at 4 °C. Interestingly, the capability of HSA to bind heme at the FA1 site renders HSA:heme functionally similar to other O_2_-transporter hemoproteins such as Hb and Mb. However HSA:heme lacks the proximal histidine residue necessary for the formation of the fifth coordination bond of the heme-Fe atom, which in Hb and Mb allows for the prosthetic group to reversibly bind O_2_. Physiological responses to exchange transfusion in acute anemia using recombinant HSA:heme revealed that this synthetic RBC substitute can resuscitate hemorrhagic shock, suggesting its promising future use as a new class of RBC substitute. Engineered HSA:heme may be a viable alternative in hemo transfusions, without the risks deriving from the transmission of pathogenic infections and incompatibilities between blood groups. In the future, further studies are required to explore this intriguing possibility.

Overall, the multifunctional properties of HSA are causing its role to be redefined beyond that of a mere plasma expander. The increasing knowledges on HSA ligand binding properties and protective roles will probably widen the therapeutic indications for this protein. In perspective, therapeutic approaches targeting heme removal via HSA will lead to interesting new concepts for the treatment of medical conditions, with a particular focus on hemolytic diseases [115,118].
